# Intra‐Operative Cholangiography With Indocyanine Green Used to Assess Bile Duct Patency in a Dog With a Ruptured Gallbladder Mucocele

**DOI:** 10.1002/vms3.70430

**Published:** 2025-06-06

**Authors:** Yujin Kim, Sungin Lee

**Affiliations:** ^1^ Department of Veterinary Surgery College of Veterinary Medicine Chungbuk National University Cheongju Republic of Korea

**Keywords:** cholangiography, indocyanine green, intraoperative, near‐infrared fluorescence, patency

## Abstract

In human laparoscopic hepatobiliary surgery, near‐infrared fluorescence (NIRF) indocyanine green (ICG) is commonly employed for intraoperative cholangiography to delineate anatomical structures; however, it is not yet used in veterinary medicine. This is the first veterinary case of ICG cholangiography used to confirm common bile duct (CBD) patency in a dog with a ruptured gallbladder mucocele (GBM). A 10‐year‐old female dog presented with lethargy and anorexia. Blood analysis revealed increased ALT, ALP, GGT, total bilirubin and C‐reactive protein levels. Ultrasonography revealed a ruptured GBM. To evaluate CBD patency during surgery, ICG 0.05 mg/kg was injected intravenously 3 h preoperatively. During cholecystectomy, real‐time NIRF image of ICG in the CBD showed a filling defect, indicating a partial obstruction within the lumen. After gentle massaging manipulation, CBD patency was confirmed using the NIRF image. No catheterisation or flushing of the CBD was required. The patient showed no relevant clinical signs of biliary stasis 5 months post‐surgery. Intraoperative ICG cholangiography efficiently and easily assessed CBD patency in real‐time. In this case, CBD patency was achieved by external manipulation with the surgeon's fingers. Therefore, catheterisation or flushing was not necessary. Since enterotomy or cholecystectomy was unnecessary, complications from the leakage of intestinal content or bile were avoided.

## Graphical abstract

This case report describes the first veterinary use of indocyanine green (ICG) cholangiography to confirm common bile duct (CBD) patency in a dog with a ruptured gallbladder mucocele. The technique, commonly used in human laparoscopic hepatobiliary surgery, was successfully employed during cholecystectomy. ICG injection revealed a partial CBD obstruction, which was resolved through gentle massage. This non‐invasive method allowed real‐time assessment of CBD patency without the need for catheterisation or flushing, potentially reducing complications associated with traditional techniques.

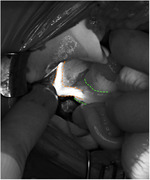

## 1 Introduction

Gallbladder mucocele (GBM) is a common gallbladder disease characterised by mucus‐filled distention of the gallbladder, which arises from secretory dysfunction. Due to the likelihood of partial or complete obstruction from mucinous material, GBM can be surgically corrected with cholecystectomy (elective or emergency surgery) (Parkanzky et al. 2019; Malek et al. 2013). Any cause of extrahepatic bile obstruction can lead to gallbladder rupture (GBR). Once GBR has occurred, confirming bile duct patency becomes crucial (Malek et al. 2013). In this context, intraoperative cholangiography proves valuable in confirming common bile duct (CBD) patency.

Cholangiography using near‐infrared fluorescence (NIRF) indocyanine green (ICG) is a useful technique in human medicine (Majlesara et al. 2017); however, it has not been practically used in veterinary medicine. Once ICG is injected intravenously, it binds to serum plasma proteins and is excreted into the bile. Through this metabolic pathway, ICG accumulates in the gallbladder and biliary tree. As ICG receives a specific wavelength of light (790–805 nm), the NIRF signal can be detected (Ris et al. 2018; Majlesara et al. 2017). Therefore, ICG cholangiography can be achieved using a certain NIRF imaging machine.

Herein, we report a case in which NIRF ICG cholangiography was used during cholecystectomy for a ruptured GBM. This technique was used to assess the CBD and confirm its patency. To our knowledge, this is the first veterinary case report of intraoperative ICG cholangiography in a patient with a ruptured GBM. Based on these results and the method's utility, we figured out that this technique was straightforward and allowed CBD assessment in this one patient.

## 2 Case Description

A 10‐year‐old, 4.385 kg, intact female Maltese presented with lethargy and anorexia. Physical examination revealed tachycardia (253 beats/min). Blood tests and ultrasonography were performed to investigate the patient. Blood chemistry analysis revealed elevated ALT (more than 2000 IU/dL, reference interval (RI): [10–118]), ALP (1946 IU/dL, RI: [20–150]), GGT (42 IU/dL, RI: [0–10]), total bilirubin (0.9 mg/dL, RI: [0.1–0.6]) and CRP (282 mg/dL, RI: [0–10]) levels. In addition, abdominal ultrasonography revealed that the internal material, suspected to be a GBM, had prolapsed from the gallbladder. The prolapsed material showed a kiwifruit‐like and stellate pattern, and the liver parenchyma close to the material showed hyperechoic echogenicity. Thus, the GBR resulting from GBM was suspected. An exploratory laparotomy was planned under these circumstances. To stabilise the patient, crystalloid fluid therapy with a maintenance rate and constant rate infusion (CRI) of fentanyl 3 µg/kg/h (Fentanyl Citrate Inj., Hana Pharm, Seoul, Korea) was administered. 0.05 mg/kg ICG (Cellbiongreen Inj., Cellbion, Seoul, Korea) was injected intravenously into the cephalic vein via an indwelling cannula 3 h before the first skin incision. For sedation, midazolam 0.2 mg/kg (Bukwang Midazolam Inj., Bukwang Pharm, Seoul, Korea) was injected intravenously. Propofol (Freepol‐MCT Inj., Daewon Pharm, Seoul, Korea) was administered to affect (2 mg/kg). After intubation, maintenance with isoflurane 1.8%–3.0% (Terrel Sol., Piramal Critical Care Inc., Bethlehem, PA, USA) and analgesia with CRI of fentanyl 1–17 µg/kg/h (Fentanyl Citrate Inj., Hana Pharm, Seoul, Korea) was conducted.

**FIGURE 1:**
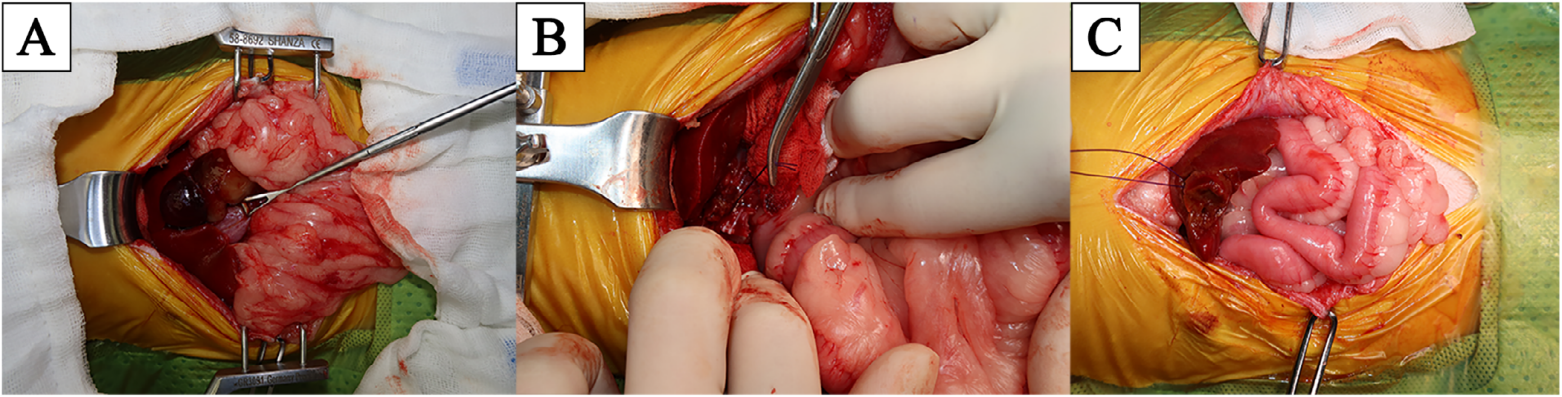
Process of exploratory laparotomy. (A) Protruded mucocele from ruptured GBM. (B) Ligation of the cystic duct with PDSII 3‐0 and two clips. (C) Left lateral liver lobe biopsy using the Guillotine method.

**FIGURE 2:**
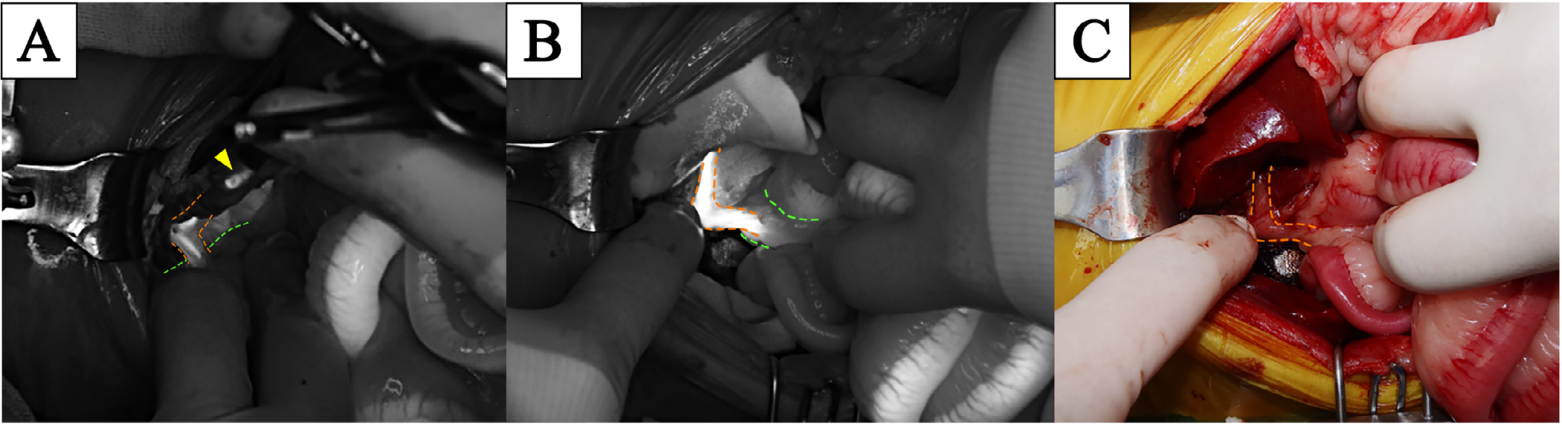
Intraoperative cholangiography of the patient with GBM. The orange dotted line indicates the hepatic duct and CBD. The green dotted line indicates the duodenum. The yellow arrowhead indicates leakage of ICG in the ruptured GB. (A) A filling defect of ICG was detected, showing a partial obstruction of the bile duct. (B) After external manipulation, the filling defect disappeared, and the patency of the CBD was confirmed. (C) Surgical view of the CBD and duodenum.

After performing a ventral medial laparotomy, the prolapsed GBM and ruptured gallbladder were identified (Figure 1A). The liver parenchyma adjacent to the gallbladder was so friable that the parenchyma tore spontaneously. After removing the protruded ball‐shaped mucocele with DeBakey forceps, suction was used to evacuate the remaining bile fluid. The ruptured gallbladder was dissected from the right medial and quadrate liver lobes using sterilised cotton swabs and electrocautery, starting from the fundus and proceeding towards the cystic duct. After gallbladder dissection (Figure 1B), ICG was detected using an NIRF imaging machine (Metagenie, MetapleBio, Korea). Under real‐time NIRF ICG, the filling defect in the CBD was easily visualised. When the surgeon gently touched the entire CBD, no firm material was palpable and the visualised filling defect appeared to be mucinous material in the CBD lumen (Figure 2A). External manipulation of the CBD was performed using real‐time ICG. The surgeon grasped the CBD with his fingers and massaged it gently, breaking the mucinous materials and moving them into the duodenum through the major duodenal papilla. The ICG‐filling defect in the CBD had resolved with ICG present throughout the entire CBD (Figure 2B,C). Due to the patency of the CBD being assessed with flow of ICG, direct CBD catheterisation either normograde via choledochotomy or retrograde via duodenotomy, was deemed necessary. After ligation of the cystic duct and artery with two non‐absorbable, polymer locking clips, cholecystectomy was performed (Fisher et al. 2022). Liver biopsy of the left lateral lobe was performed using a guillotine suture (Figure 1C). Abdominal lavage with warm Hartmann's solution greater than 200 mL/kg was conducted, followed by Jackson‐Pratt drain placement. Conventional closure of the abdomen, subcutaneous sutures and skin sutures were performed.

**FIGURE 3:**
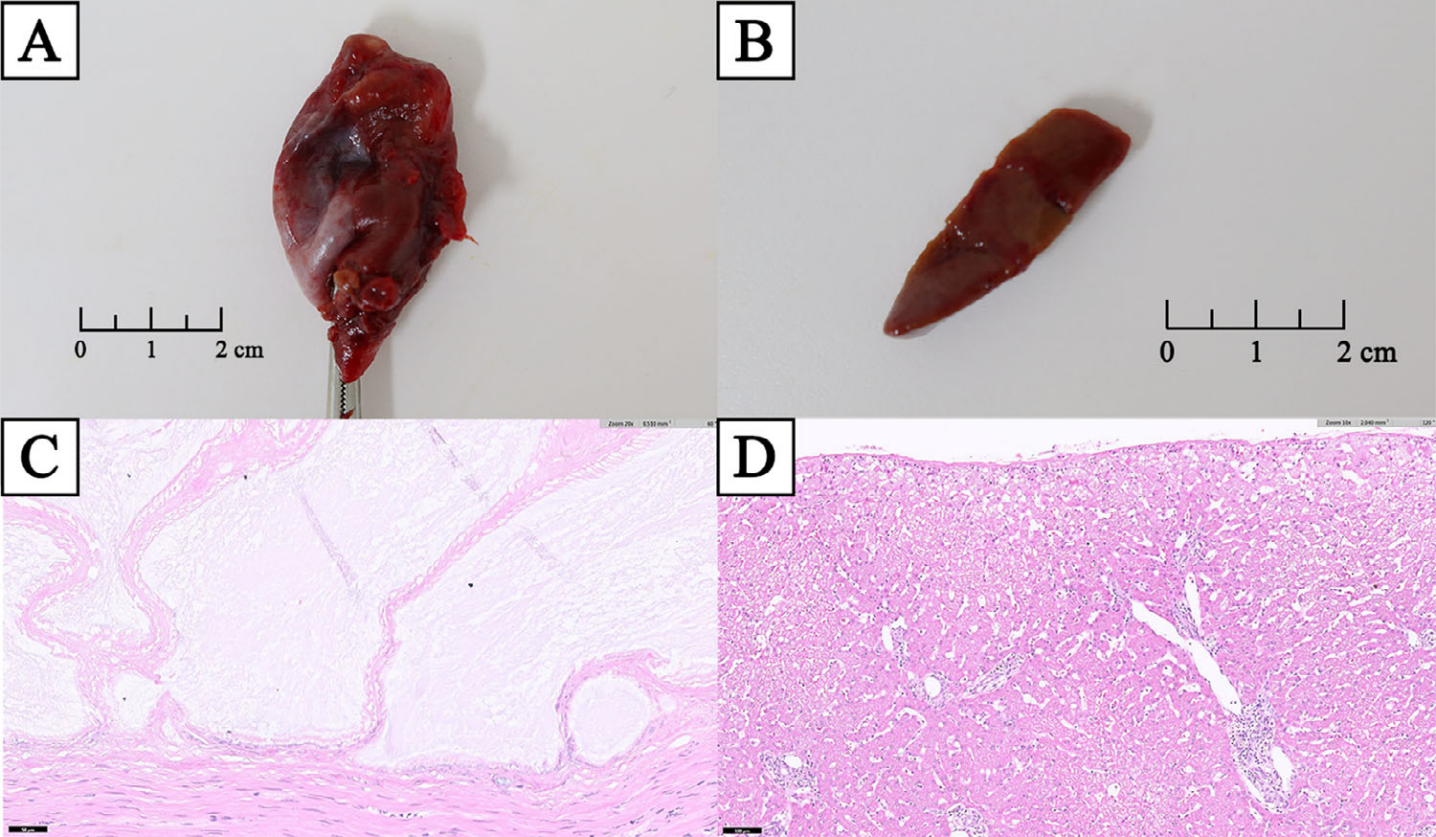
Resected tissue and histopathology of GB and liver biopsy. (A) Ruptured GB. (B) Liver biopsy of the left lateral liver lobe. (C) In the GB tissue slide, mucosal proliferation, multifocal mucosal ulceration, granulation tissue, accumulated luminal mucins, proteinaceous fluid, haemorrhage, and inflammation were observed. (D) In the liver tissue slide, lymphoplasmacytic and neutrophilic hepatitis, multifocal bile plug formation, bile stasis, biliary proliferation, and intrahepatocytic pigmentation, with extensive neutrophilic and fibrinous peritonitis, were observed.

Histopathological analyses of the excised gallbladder and liver biopsy samples were performed (Figure 3A,B). The gallbladder showed hyperplastic mucosal proliferation and multifocal mucosal ulceration, which is consistent with ruptured GBM (Figure 3C). The liver showed lymphoplasmacytic and neutrophilic hepatitis, multifocal bile plug formation, bile stasis, biliary proliferation and intrahepatocytic pigmentation, with extensive neutrophilic and fibrinous peritonitis (Figure 3D).

The patient was monitored and hospitalised for 3 days, and showed progressive improvement with no clinical signs attributable to ongoing biliary tract obstruction. Serum total bilirubin level was normalised (0.3 mg/dL). At the most recent follow‐up 5 months after surgery, the dog did not show vomiting, anorexia, lethargy, icterus or other signs of biliary obstruction or bile leakage.

## 3 Discussion

Cholangiography in veterinary medicine has relied on modalities such as fluoroscopy (Kanai et al. 2022), computed tomography (CT) and magnetic resonance imaging (MRI) (Marolf 2016). However, CT and MRI should be conducted perioperatively as they cannot provide real‐time monitoring of CBD patency or changes in the biliary tree structure throughout the surgery. Conventional C‐arm‐assisted cholangiography using iodixanol is performed intraoperatively; however, it shows a radiographic image rather than a real surgical view. There is a potential risk of radiation exposure (Kanai et al. 2022; Marolf 2016). To overcome these limitations, we used ICG‐NIRF cholangiography.

In human medicine, intraoperative ICG‐NIRF cholangiography has been used, especially in hepatobiliary surgery, to confirm anatomic structures such as Calot's triangle (Symeonidis et al. 2024; Majlesara et al. 2017; Aoki et al. 2010). According to human studies, ICG cholangiography‐guided surgery has a shorter operation time (Aoki et al. 2010) and improved visualisation of the CBD and common hepatic duct (Symeonidis et al. 2024). Moreover, it does not require radiation or additional intraoperative procedures. It generates real‐time visualisation and immediately reflects the surgeon's manipulation. In our case, we injected ICG intravenously before surgery; hence, there was no need for another step to visualise the CBD. Moreover, the surgeon's external manipulation to push the mucinous materials into the duodenum was performed under ICG cholangiography, and the CBD patency was confirmed in real time. There was no need for catheterisation or flushing of the CBD owing to the immediate confirmation of CBD patency; therefore, surgery time was reduced and other procedures such as duodenotomy were avoided.

Trials using ICG cholangiography in dogs have also been reported (Kim and Lee 2024; Larose et al. 2024). In a pilot study, ICG cholangiography was performed in purpose‐bred dogs, which provided improved visualisation of the biliary tree (Larose et al. 2024). Although ICG cholangiography has many advantages and successfully visualised the biliary tree in this pilot study, it has not been used practically in a patient with hepatobiliary disease. We used intraoperative ICG cholangiography in ruptured GBM patients based on the pilot study's dose and time data (0.05 mg/kg, 3 h before surgery). This method can easily identify biliary structures, especially the CBD. There was also no problem confirming the CBD patency owing to the intuitive fluorescent image of ICG.

Furthermore, we attained CBD patency using external manipulation only. Generally, flushing or catheterisation using the normograde or retrograde method is performed to secure CBD patency in patients with GBRs (Putterman et al. 2021). To flush or insert a stent within the CBD, normograde approach via choledochotomy or retrograde approach via duodenotomy should be performed, and these procedures are known to occasionally cause complications such as bile leakage and peritonitis, dehiscence at the CBD or duodenotomy site, obstruction of the choledochal stent, dislodgement or migration of the stent, ascending cholangiohepatitis, and severe local inflammation caused by the stent material (Mehler 2011; Claeys 2016). A retrospective study demonstrated a risk of bile leakage in 7% of dogs without intraoperative cholangiography owing to manipulation of the gallbladder. To confirm the CBD patency, catheterisation was performed in one dog in a previous study; however, clinical signs recurred 1 month post‐operatively (Malek et al. 2013). Another study comparing the prognosis of normograde or retrograde flushing showed that, although the incidence rate differed between flushing directions, there were common complications, such as postoperative pancreatitis, bile peritonitis or persistent CBD obstruction (Putterman et al. 2021). In this case, however, we confirmed the CBD patency without any incision (choledochotomy or duodenotomy) but with gentle manipulation to move the mucinous material from the lumen of the CBD to the duodenum through the major duodenal papilla. As mentioned, real‐time external manipulation of the CBD under ICG cholangiography to confirm patency was successfully performed in this case, without an invasive approach to the biliary tree or intestine.

There have been long discussions on whether CBD should be flushed in patients with GBM. Some surgeons advocate flushing the CBD in all patients with GBM (Kanai et al. 2022), whereas others insist that there is no difference in outcomes between the flush and non‐flush groups (Hernon et al. 2023). However, in that study, the population of patients with GBR within the flush and non‐flush groups (two dogs in each group) cannot provide definitive evidence to decide whether to flush the CBD in case of GBR or full obstruction in the extrahepatic biliary system is strongly suspected. And the others have asserted that flushing or catheterisation should be performed only when biliary obstruction is confirmed (Piegols et al. 2021). These arguments have been ongoing as no clear scientific evidence has been established so far; thus, through intraoperative ICG cholangiography, CBD patency can be confirmed in real‐time and visualised images of the CBD can provide scientific evidence to decide whether to flush the CBD. Based on this evidence, we decided not to flush the CBD, and there were no further complications related to biliary obstruction in this one patient.

In conclusion, intraoperative ICG cholangiography is a real‐time and convenient method for CBD patency visualisation in a ruptured gallbladder with GBM. This can help surgeons decide whether to flush the CBD. Intraoperative ICG cholangiography provides objective evidence that reflects the real‐time state of the bile duct and can be routinely used during open or laparoscopic cholecystectomy, as in humans.

## Author Contributions

**Yujin Kim**: conceptualisation (equal), data curation (equal), formal analysis (equal), investigation (lead), methodology (equal), validation (equal), visualisation (equal), writing – original draft (lead), writing – review and editing (equal). **Sungin Lee**: conceptualisation (equal), data curation (equal), formal analysis (equal), funding acquisition(lead), investigation (supporting), methodology (equal), supervision (lead), validation (equal), visualisation (equal), writing – original draft (supporting), writing – review and editing (equal)

## Ethics Statement

The authors have nothing to report.

## Consent

Written informed consent for publication of the clinical details was obtained from the dog's owner.

## Conflicts of Interest

The authors declare no conflict of interest to declare.

## Peer Review

The peer review history for this article is available at https://www.webofscience.com/api/gateway/wos/peer‐review/10.1002/vms3.70430.

## Data Availability

Data sharing not applicable to this article as no datasets were generated or analysed during the current study.
